# On Being the Right Size as an Animal with Plastids

**DOI:** 10.3389/fpls.2017.01402

**Published:** 2017-08-17

**Authors:** Cessa Rauch, Peter Jahns, Aloysius G. M. Tielens, Sven B. Gould, William F. Martin

**Affiliations:** ^1^Molecular Evolution, Heinrich-Heine-University Düsseldorf, Germany; ^2^Plant Biochemistry, Heinrich-Heine-University Düsseldorf, Germany; ^3^Department of Biochemistry and Cell Biology, Faculty of Veterinary Medicine, Utrecht University Utrecht, Netherlands; ^4^Department of Medical Microbiology and Infectious Diseases, Erasmus University Medical Center Rotterdam, Netherlands

**Keywords:** *Elysia*, Sacoglossa, photosynthetic slugs, photosynthetic animal, growth rate, life cycle

## Abstract

Plastids typically reside in plant or algal cells—with one notable exception. There is one group of multicellular animals, sea slugs in the order Sacoglossa, members of which feed on siphonaceous algae. The slugs sequester the ingested plastids in the cytosol of cells in their digestive gland, giving the animals the color of leaves. In a few species of slugs, including members of the genus *Elysia*, the stolen plastids (kleptoplasts) can remain morphologically intact for weeks and months, surrounded by the animal cytosol, which is separated from the plastid stroma by only the inner and outer plastid membranes. The kleptoplasts of the Sacoglossa are the only case described so far in nature where plastids interface directly with the metazoan cytosol. That makes them interesting in their own right, but it has also led to the idea that it might someday be possible to engineer photosynthetic animals. Is that really possible? And if so, how big would the photosynthetic organs of such animals need to be? Here we provide two sets of calculations: one based on a best case scenario assuming that animals with kleptoplasts can be, on a per cm^2^ basis, as efficient at CO_2_ fixation as maize leaves, and one based on ^14^CO_2_ fixation rates measured in plastid-bearing sea slugs. We also tabulate an overview of the literature going back to 1970 reporting direct measurements or indirect estimates of the CO_2_ fixing capabilities of Sacoglossan slugs with plastids.

## Introduction

**Figure 1:**
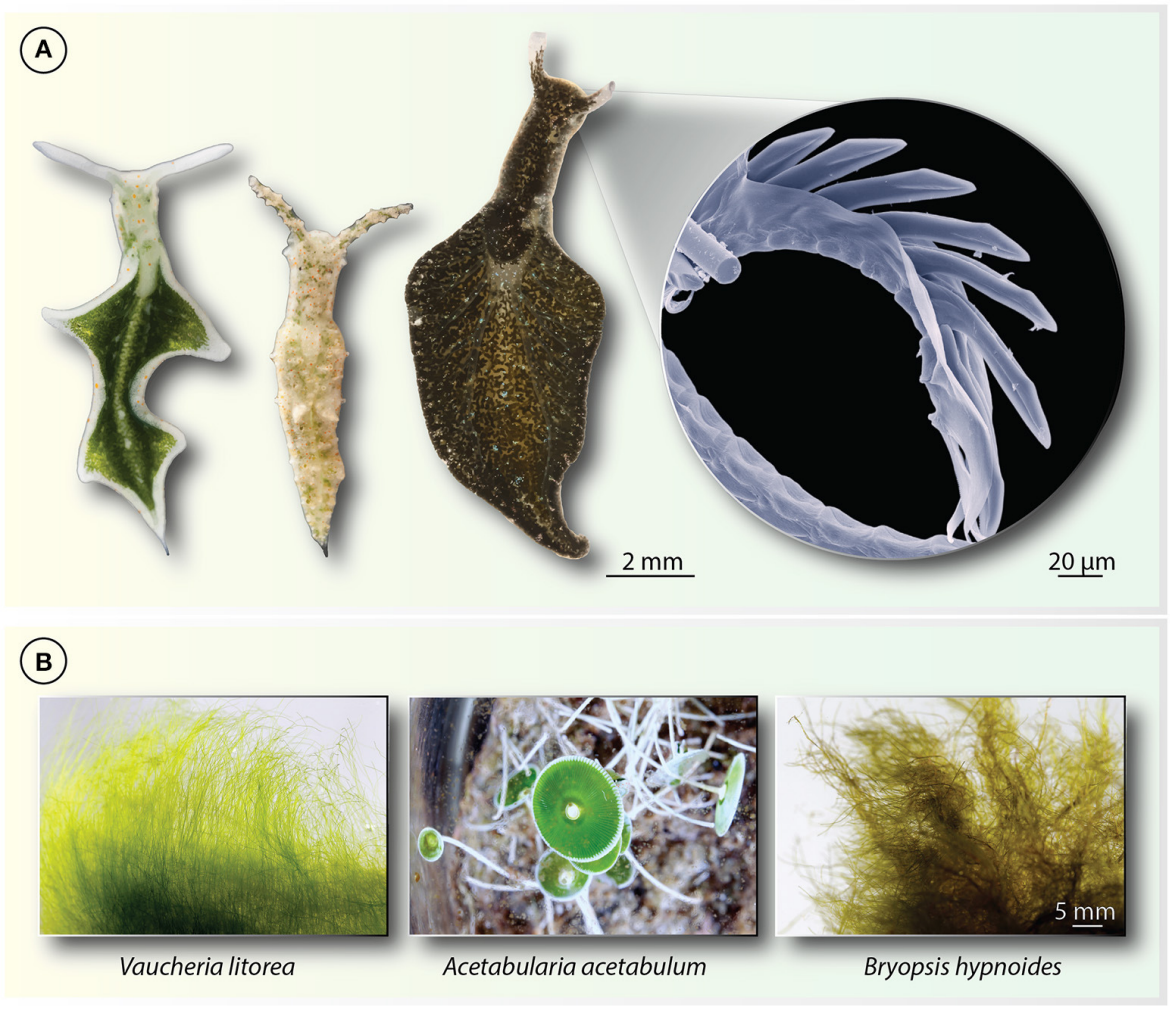
Examples of sacoglossan sea slugs and their algal food. **(A)** From left to right the three Sacoglossa *Elysia timida, Elysia cornigera*, and *Elysia viridis*. All use a radula to penetrate siphonaceous algae from which they suck out the cytosolic content (hence the name sap sucking slugs) that includes compartments such as mitochondria, nuclei, and plastids. Plastids are specifically sequestered (the kleptoplasts) and incorporated into the cells that line the digestive tubules, in some cases giving the slugs their characteristic green color. The radula is a chitinous feeding structure of molluscs comparable teeth. A scanning-electron micrograph of one of *E. viridis* is shown on the very right, revealing the individual teeth-like structures the animals use to penetrate the algal cell wall. **(B)** Examples of macroalgae on which Sacoglossa feed and that are siphonacous. Some Sacoglossa are highly specialized and feed on single alga species, such as *E. timida* that only feeds on *Acetabularia*, while others such as *E. viridis* can feed on a larger variety of macroalgae.

The group of sea slugs belonging to the order Sacoglossa really know how to keep biologists busy. The group comprises about 400 species of small, soft-bodied marine animals (Figure 1A) that feed upon algae (Jensen, 2007; Wägele et al., 2011). The algal food is what mainly keeps the biologists busy. Adult sacoglossans feed upon not just any algae, but upon siphonaceous algae (Figure 1B). Siphonaceous describes large, tube-like filamentous algae, often having a reduced, hardly visible vacuole, meaning that they are filled with a rich cytosol, sometimes containing many hundreds of plastids. The sacoglossans possess a highly-specialized radula, a molluscan version of teeth (Figure 1A; zoom-in panel), that allows them to puncture their algal prey and suck out the cytoplasmic contents, which they then digest (Trench, 1969; Wägele et al., 2011).

A small number of sacoglossan species perform a very special trick: they do not digest the plastids, but they sequester them instead as kleptoplasts within the cells of their digestive gland (Rumpho et al., 2001; Händeler et al., 2009; de Vries et al., 2014a), giving the slugs their distinct green color. Electron microscopy experiments showed that the plastids can stay morphologically intact for days, weeks, or even months (Greene, 1970; Rumpho et al., 2001; Händeler et al., 2009). Early investigations in the 1970s indicated that the sequestered plastids remain photosynthetically active (Giles and Sarafis, 1972; Hinde and Smith, 1972; Trench and Ohlhorst, 1976). Accordingly, these sacoglossans have been described as “photosynthetic” for many decades. Indeed, the slugs could survive in the light for weeks without food, just with plastids (Pierce et al., 1996). More recent investigations, however, showed that the slugs with plastids survive in the dark, too, and that the presence of photosynthesis inhibitors also does not significantly, if at all, increase the rate with which they lose weight (Christa et al., 2014; de Vries et al., 2015). It seems that some sacoglossan species are simply more starvation tolerant than others and that has to do with better coping with reactive oxygen species (de Vries et al., 2015). Indeed, all slugs shrink during starvation and some can go from centimeters to millimeters in length when deprived of a food source. The green sacoglossans are no exception if starved (Mujer et al., 1996; Pelletreau et al., 2012; Klochkova et al., 2013; Christa et al., 2014; de Vries et al., 2015), that is, if they are forced to turn to autophagy for energy generation (de Vries et al., 2015). It thus appears that what we once thought were animals that are good at photosynthesis are first and foremost good at fasting. However, the concept of “photosynthetic animals” has considerable inertia in the literature. The media in particular regularly want to know about the status and future of photosynthetic animals.

Such discussions inevitably lead to debates of whether it might someday be possible to construct photosynthetic farm animals, given all that modern science can do with gene technology and the like. Thus, there is a need to critically inspect the concept of photosynthetic animals. The first and most obvious issue is that they would need a lot of surface area to harvest that light (Smith and Bernays, 1991). How much area would they need and, conversely, what portion of a green slug's food requirements can be covered from sequestered plastids? In the spirit of Haldane et al. (1926) essay “On being the right size,” let us take a look, using some rough but robust and very conservative estimates.

## The size of photosynthetic animals

If we go into the literature, we can readily find most of the values that we need. Fast growing dairy cows gain weight at a rate of about 1 kg per day, slow growing cows gain roughly 0.5 kg per day (Brody and Ragsdale, 1930). Let's use the 0.5 kg per day value, to which we will return later. Another value we need is how much food is required to generate that weight. An old rule of thumb is that roughly 5–6 kg of maize are needed to generate 1 kg of beef, though modern numbers provide a range of 3–10 kg of maize per kg of beef (Tilman et al., 2012). Cows consist of more than beef, though, so let's be generous and assume that only 3 kg of maize are needed per kg of cow bodyweight. In order to get our cow through one growing season, that is, through 1 year (and hence 180 kg of weight gain), 540 kg of maize would be required—let's grant another 10% in improved feed conversion efficiency and call it 500 kg of maize seed that is needed to grow our cow for a year. This is deliberately an underestimate, to be conservative.

How much leaf area is needed to produce those 500 kg of maize seed? Under excellent field conditions (good soil, good sunlight, sufficient water, and fertilizer) maize can yield about 160 bushels per acre (http://cropwatch.unl.edu/corn/yieldtrends), which converts to about 10 tons per hectare, or roughly 1 kg/m^2^. Maize has a leaf area index of about 3, meaning 3 m^2^ leaf area per m^2^ field area (Watson, 1947; Wilhelm et al., 2000), such that yield, expressed in units of leaf area, corresponds to roughly 0.3 kg of maize seed per square meter of maize leaf area. Since we need 500 kg of maize per season, that means that our cow would need about 1,600 m^2^ of surface area with the photosynthetic efficiency of maize (Tollenaar, 1983), one of the top-efficiency photosynthesizers known, to sustain that 0.5 kg per day growth rate, averaged over the year. Thus, under optimal field conditions and with the photosynthetic efficiency of maize, our photosynthetic cow needs a leaf roughly 40 × 40 m in size, about a third the size of a football field. We note that unless our leaf-bearing cow has the phenotypic appearance of a maize plant, it is going to have a leaf area index very close to 1, which is 3-fold less efficient per square meter than maize, even if it has the same photosynthetic efficiency.

Of course, our estimate so far assumes that the biogenesis and maintenance of the photosynthetic organ itself consumes no photosynthates. Were the organ is on average 1 cm thick, it would weigh 10 kg/m^2^, which corresponds to 16,000 kg total “leaf” weight because we need 1,600 m^2^ of photosynthetic surface area to generate 500 kg of maize seed to support 180 kg of weight gain per year (growing season). That is going to pose insurmountable problems, because the cow's “leaf” requires 88 times more body mass synthesis than the 180 kg of weight gain it is supposed to support. So let us assume that molecular biologists and gene engineers can find ways to make it 1 mm thick on average, adding only 1,600 kg of extra weight. That leaf will require 9 growing seasons to synthesize at a growth rate of 180 kg per year, and we have still not generated the 180 kg per year net surplus cow material for harvesting. Thirty months is a late “terminal age” for cattle (Bouton et al., 1978), meaning our photoengineered cow would have to spend three lifetimes growing its leaf before it gets started growing the body (at 180 kg per year). It is rapidly becoming clear that the photosynthetic cow project (a photobovid) is not going to work out very well, and we furthermore are beginning to understand more clearly why cows eat grass all day, leaving it to plants to do the heavy lifting of photosynthesis and leaving it to their anaerobic gut flora to break cellulose down into short chain fatty acids (acetate, propionate, and butyrate) that the cows can resorb as food (Bauchop and Mountfort, 1981; Dijkstra et al., 1993).

Let's be unrealistically generous, however, and say that our cow does not have to invest any photosynthates at all in the synthesis of its leaf—it gets the leaf for free. This would then be equivalent to the situation of the saccoglossan slugs, which grow up without photosynthesis and incorporate plastids into a ready-made leaf, their parapodia. If we generously gauge the shadow-generating surface area of the cow without the leaf as 2 m^2^, then the photosynthesizing animal needs to have an organ with 800 times its own photosynthetically effective surface area. Put another way, with its own surface, the animal could cover about 0.1% of its growth needs (2/1,600), if it was, on a per cm^2^ basis, as photosynthetically efficient as maize.

We have also left another important limiting factor—water demands—out of the calculation. The situation at the water trough for those engineered farm animals opens up a whole new set of problems into which we do not delve here. That brings us back to the slugs. The slugs are different from the cows, of course. They are a lot smaller, they are not warm-blooded so they do not need to generate lost heat, and they have a different growth rate.

## The plastid contribution to physiology in slugs

A well-studied slug species is *Elysia timida* (Figure 1A), which under good laboratory conditions can grow to a fresh weight of roughly 100 mg, corresponding to a dry weight of ca. 10 mg per animal. In our own *E. timida* laboratory cultures and controlled conditions (Schmitt et al., 2014), the time from egg hatching to the juvenile stage is about 20 days. They start feeding on siphonaceous algae at day 3 after egg hatching (Schmitt et al., 2014). By day 20 they are ~0.1 mg in weight and 0.5 mm long. From that point, they require an additional 7–8 weeks to reach their final size with a dry weight of roughly 10 mg. In total, it takes about 12 weeks for *E. timida* to develop from eggs to maturity (Schmitt et al., 2014).

How much of that dry weight can come from photosynthesis in sequestered plastids? The average eukaryotic cell has a dry weight formula of roughly C_5_H_7_O_2_N, meaning that eukaryotes are roughly 50% carbon by dry weight (Heldal et al., 1985). A slug of ca. 10 mg dry weight therefore contains 5 mg of carbon (5 mg C). From Christa et al. (2014) we know that the experimentally determined CO_2_ incorporation rates for *E. timida* are 30 nmol of CO_2_ in 2 h for four animals. This translates to 45 nmol C per animal per 12-h photosynthetic period, which converts to 540 ng of carbon per animal per 12-h-daylight day. The adult animal contains 5 mg C; photosynthesis in fully grown animals can provide 0.54 μg per day, which corresponds to 0.011% incorporation of the final C content per day. If that rate were to continue for 84 days (the development period) the slug could, theoretically, fix 0.91%, or about 1% of its carbon.

Being able to fix 1% of one's carbon is not very photosynthetic one might say, but 1% is still a far too generous estimate, we need to correct for at least two more factors. First, the young adults are only about 500 μm long with an area of 0.25 mm^2^ (Schmitt et al., 2014), or 0.3% the area of the fully-grown animal, which has a plastid-containing surface area of roughly 1 cm^2^ (Figure 1A). Over the 84-day development period, on average half the surface area of the adult is available for photosynthetic activity, so that perhaps 0.5% of the animal's C can be contributed by photosynthesis.

But a contribution of 0.5% total C from photosynthesis to slug body weight is still too generous, because there is also the issue of carbon turnover. That is, the animals respire some of the C that they assimilate, whether from heterotrophic feeding (which is already >99% of total C, as we see from our calculation so far), or from the plastid contribution. The turnover time, or half-life, of C has not been measured so far in *E. timida*. There are, however, very good numbers available for C half-life in animals of many different sizes (van der Zanden et al., 2015), where it is seen that C-turnover times in animals scale tightly with body size. From the published curves of van der Zanden et al. (2015), we can estimate that in the case of invertebrates that weigh as much as *E. timida*, the C half-life should fall in the range of 12 days. That means that at constant weight, half of the carbon atoms present in the animal at time zero are still present after 12 days. But we do not have to worry too much about the carbon half-life, because the measured value of 540 ng C per animal per 12-h day is net incorporation, it already takes respiration into account. In the 12 h of dark, there is still respiration, so that the value of 0.5% of the animal's C needs to be halved once more (continued respiration but no photosynthesis in the 12-h dark phase) and we arrive at an estimate that roughly 0.25% of the adult's C can be contributed by photosynthesis, based on the most recent experimentally measured ^14^CO_2_−based CO_2_ incorporation rates for slugs available in the literature (Christa et al., 2014).

Thus, *E. timida* might be able to cover about 0.25% of its body weight increase during development from CO_2_ fixation in plastids. That explains why some plastid-harboring sacoglossan slugs have been observed to lose weight in the light at the same rate as in the dark or with chemically inhibited photosynthesis (Christa et al., 2014).

However, as in any field of active scientific inquiry, there are conflicting reports, of course. For example, Laetz et al. (2017) recently reported that starch provided by plastids increases in *E. timida* during starvation before it decreases, and that this impacts slug survival. Those are important observations, but quantitative estimates of CO_2_ fixation based on ^14^CO_2_ incorporation were lacking. Earlier reports of carbon fixation by plastids in sacoglossans based on stable carbon isotope ratios (^13^C/^12^C) gave very high estimates of net photosynthetic contribution by slug-sequestered plastids (Raven et al., 2001), but quantitative estimates of CO_2_ fixation based on ^14^CO_2_ incorporation were again lacking. Some of the earliest ^14^CO_2_ incorporation measurements from the 1970s delivered fairly high values (Hinde and Smith, 1975), but were also marked by considerable variation from measurement to measurement and in at least one case more ^14^CO_2_ incorporation measured in the dark than in the light.

When we embarked upon measuring ^14^CO_2_ incorporation in slugs, we also noticed disconcerting variation across experiments and puzzlingly high dark ^14^CO_2_ incorporation values (AGMT and WFM, unpublished observations). We subsequently found that prolonged (overnight) and very strong acid treatment (1 M HCl) was needed to gasify and purge unincorporated ^14^CO_2_ from animal homogenates after incubation experiments. If photosynthesis is the reason that sacoglossan slugs keep plastids, then we should be able to quantify that contribution via ^14^CO_2_ incorporation measurements in other species that, like *E. timida*, sequester plastids.

## Looking into the literature: expectations and evidence

If we dig into the literature on the possible function of photosynthesis in plastids during starvation in Sacoglossa, it is apparent that only very few studies carried out ^14^CO_2_ incorporation measurements that would allow one to directly determine CO_2_ incorporation (Table 1). Furthermore, many studies lacked the appropriate controls that would permit a clear causal connection between photosynthetic CO_2_ fixation and animal survival, for example testing the effect of chemical inhibition of photosynthesis. There is a crucial difference between (a) the inference that sequestered, morphologically intact, plastids are important for enduring starvation (for possibly unknown reasons), and (b) the inference that sequestered, morphologically intact, plastids are providing CO_2_ fixation at rates that would provide significant nutrition to the animals. No previous work, nor the present calculations at hand, call into question the view that sequestered plastids in long term retention Sacoglossa have some kind of biological significance. But we are also not aware of any previous work that experimentally justifies interpretations or claims that the quantity of fixed carbon provided by plastids is sufficient to support the idea of a “photoautotrophic animal,” as pervades the literature on plastid-sequestering Sacoglossa, not seldom in the title or abstract. Our present calculations serve to underscore the point that measured ^14^CO_2_ fixation rates for plastids sequestered in the cytosol of an animal cell cannot support animal growth.

**Table 1:** Overview of relevant literature on Sacoglossa and experimental details.

**References**	**# spec. (1)**	**# indv. (2)**	**wgt/len (3)**	**^14^CO_2_ (4)**	**Fv/Fm (5)**	**dark ctrl. (6)**	**DCMU (7)**	**Chl. (8)**	**Starved (9)**	**O_2_ (10)**
Trench, 1969	1			+		+		+	+	+
Greene, 1970	2	8		+		+		+	+	
Trench et al., 1970	1			+						
Taylor, 1971	1	6						+		+
Greene and Muscatine, 1972	3	11		+		+		+		
Hinde and Smith, 1972	1	39	+	+		+		+	+	
Trench et al., 1973	1			+		+		+	+	
Gallop, 1974	1			+				+	+	
Hinde and Smith, 1974	4	68	+	+		+		+	+	
Trench et al., 1974	2			+						
Hinde and Smith, 1975	1		+			+		+	+	
Kremer and Schmitz, 1976	1			+						
McLean, 1976	1								+	
Trench and Ohlhorst, 1976	2			+						
Clark and Busacca, 1978	4		+					+	+	
Gallop et al., 1980	1	27	+	+		+		+	+	
Clark et al., 1981	1			+		+		+	+	
Weaver and Clark, 1981	5									
Marín and Ros, 1989	4		+	+				+	+	
de Freese and Clark, 1991	3	24		+		+				
Marín and Ros, 1992	1		+	+						
Marín and Ros, 1993	1		+							
Mujer et al., 1996	1	220	+						+	
Green et al., 2000	1	25				+		+	+	+
Wägele and Johnsen, 2001	4	10			+					
Cueto et al., 2005	1									
Casalduero and Muniain, 2006	1	184	+						+	+
Curtis et al., 2006	1	5							+	
Evertsen et al., 2007	7	17			+				+	
Casalduero and Muniain, 2008	1	357	+			+		+		+
Rumpho et al., 2008	1									
Evertsen and Johnsen, 2009	2		+		+			+	+	
Händeler et al., 2009	29	186			+				+	
Pierce et al., 2009	1							+	+	
Jesus et al., 2010	1	20			+	+		+	+	
Maeda et al., 2010	18									
Schwartz et al., 2010	1								+	
Pierce et al., 2011	1								+	
Wägele et al., 2011	2	16			+				+	
Devine et al., 2012	1	45							+	
Pelletreau et al., 2012	1	80	+						+	
Bhattacharya et al., 2013	1								+	
Christa et al., 2013	1	41			+	+			+	
de Vries et al., 2013	1									
Klochkova et al., 2013	1	400	+		+	+		+	+	
Baumgartner et al., 2014	1		+							
Pelletreau et al., 2014	1		+			+			+	
Schmitt et al., 2014	1	179			+				+	
Serôdio et al., 2014	3		+		+					
Baumgartner et al., 2015	1		+		+					
Christa et al., 2015	105				+	+			+	
Cruz et al., 2015	2				+	+		+		
Curtis et al., 2015	3				+				+	
de Vries et al., 2015	2		+	+	+	+	+	+	+	
Martin et al., 2015	4							+		
Laetz et al., 2017	1	40	+		+		+		+	
Wade and Sherwood, 2017	1	69			+				+	

*The switch in methods used in particular regarding CO_2_ fixation experiments and using pulse amplitude modulation (PAM) measurements (i.e., Fv/Fm ratios). Columns from left to right: (1) number of different species reported; (2) total number of slugs used; (3) weight (wgt) and/or length (len) measured; (4) ^14^CO_2_ incorporation studies; (5) photosynthetic activity measured by means of Pulse Amplitude Modulation (PAM) measurements; (6) group of slugs was also kept in the dark; (7) chemical blocking of photosynthesis by DCMU; (8) chlorophyll/pigment concentration measurements; (9) a group of slugs were starved throughout the experiments; (10) O_2_ evolution was measured*.

## Conclusion

The purpose of this paper is to provide a reference to which one might turn in the event that the media or an interested high school class calls, wanting to know whether the engineering of photosynthetic farm animals “like the slugs” might be a worthwhile avenue of scientific pursuit. The answer we obtain is that by weight, about 0.3% of the slug might be able to survive from photosynthetic activity through its sequestered plastids. The other 99.7% has to live from normal ingested food, like the rest of us animals. Somewhat more bleak prospects arise for photosynthetic farm animals, because of their size and warm-blooded nature. In principle, the concept of photosynthetic animals is interesting. In practice, it underscores the observation that among all animals known so far; only seven species of sacoglossan slugs steal plastids from siphonaceous algae and sequester them long-term (de Vries et al., 2014b), apparently for reasons other than carbon fixation.

## Author contributions

CR, SG, and WM drafted the MS. CR designed the figure and the table. CR, PJ, AT, SG, and WM all made a substantial, direct and intellectual contribution to the work, and approved it for publication.

### Conflict of interest statement

The authors declare that the research was conducted in the absence of any commercial or financial relationships that could be construed as a potential conflict of interest. The reviewer BML and handling Editor declared their shared affiliation, and the handling Editor states that the process met the standards of a fair and objective review.
